# A truncated Kv1.1 protein in the brain of the *megencephaly *mouse: expression and interaction

**DOI:** 10.1186/1471-2202-6-65

**Published:** 2005-11-23

**Authors:** Ann-Sophie Persson, Göran Klement, Malin Almgren, Kristoffer Sahlholm, Johanna Nilsson, Susanna Petersson, Peter Århem, Martin Schalling, Catharina Lavebratt

**Affiliations:** 1 Neurogenetic Unit, Department of Molecular Medicine and Surgery, CMM, Karolinska Hospital, Karolinska Institutet, 171 76 Stockholm, Sweden; 2 Nobel Institute for Neurophysiology, Department of Neuroscience, Karolinska Institutet, Stockholm, Sweden; 3 The Ludwig Institute for Cancer Research, Stockholm Branch, Stockholm, Sweden

## Abstract

**Background:**

The megencephaly mouse, *mceph/mceph*, is epileptic and displays a dramatically increased brain volume and neuronal count. The responsible mutation was recently revealed to be an eleven base pair deletion, leading to a frame shift, in the gene encoding the potassium channel Kv1.1. The predicted MCEPH protein is truncated at amino acid 230 out of 495. Truncated proteins are usually not expressed since nonsense mRNAs are most often degraded. However, high *Kv1.1 *mRNA levels in *mceph/mceph *brain indicated that it escaped this control mechanism. Therefore, we hypothesized that the truncated Kv1.1 would be expressed and dysregulate other Kv1 subunits in the *mceph/mceph *mice.

**Results:**

We found that the MCEPH protein is expressed in the brain of *mceph/mceph *mice. MCEPH was found to lack mature (Golgi) glycosylation, but to be core glycosylated and trapped in the endoplasmic reticulum (ER). Interactions between MCEPH and other Kv1 subunits were studied in cell culture, *Xenopus *oocytes and the brain. MCEPH can form tetramers with Kv1.1 in cell culture and has a dominant negative effect on Kv1.2 and Kv1.3 currents in oocytes. However, it does not retain Kv1.2 in the ER of neurons.

**Conclusion:**

The *megencephaly *mice express a truncated Kv1.1 in the brain, and constitute a unique tool to study Kv1.1 trafficking relevant for understanding epilepsy, ataxia and pathologic brain overgrowth.

## Background

The megencephaly mice (BALB/cByJ-*Kv1.1*^*mceph*/*mceph*^, here denoted *mceph/mceph*) [1] have an 11 base pair deletion in the *Shaker*-like voltage-gated potassium channel subunit *Kv1.1 *[2]. This mutation causes progressive postnatal complex partial seizures and a unique pathologic brain overgrowth [2]. The enlargement is not uniform but restricted to the hippocampus and ventral cortex, with 28% and 72% larger area compared to wildtype at 12 weeks of age [3]. The enlargement is in part due to that the numbers of both neurons and glia cells are dramatically increased in the hippocampus which is caused by increased proliferation and/or reduced apoptosis (Almgren et al, unpublished).

The 11 base pair deletion in Kv1.1 leads to a frame shift and a premature stop codon. The predicted truncated Kv1.1 protein (MCEPH) will retain only the N-terminal (T1) domain, the first transmembrane domain (S1) and the first extracellular loop. Voltage gated potassium channels form a diverse group of membrane proteins, regulating membrane potential, neuronal excitability and nerve signaling [4]. The channels are hetero- or homotetramers, formed by a great variety of subunits, classified in 12 subfamilies [5]. Kv1.1 belongs to the Kv1 subfamily, consisting of eight members (Kv1.1 to Kv1.8). Kv1.1, Kv1.2 and Kv1.4 are the most abundant Kv1 subunits expressed in the brain [6]. Although the heteromeric structure means that a very large number of K channels can be formed in theory [7,8], the composition of the heteromultimers in the mammalian brain seems to be precisely regulated [9,10]. A direct link between potassium channel dysfunction and apoptosis is that reduced intracellular potassium levels appear to promote critical events early in the suicide program. Treatment with potassium channel blockers have been shown to block apoptosis in various cell types [11]. However, neither *Kv1.1 *mutations, the Kv1.1 null mouse [12] nor other epileptic models have previously been associated with pathologic brain overgrowth. Therefore, we hypothesized that the truncated Kv1.1 would be expressed and dysregulate other Kv1 subunits in the *mceph/mceph *mice.

Truncated proteins are usually not expressed. This is because mRNAs with a premature stop codon are degraded through nonsense-mediated mRNA decay (NMD) [13]. One exception to this is genes with no introns such as the *Kv1 *genes [14]. In *mceph/mceph *mice *in situ *hybridization have shown that there is no decay but instead an increased expression of *Kv1.1 *mRNA in the hippocampus, cortex and ventral cortex [2]. Thus, it is possible that MCEPH is expressed.

In humans *Kv1.1 *point mutations are reported in patients with the autosomal dominant disorder episodic ataxia type1 (EA1), [15]. Most are missense mutations but there is one report of a premature stop codon, resulting in a Kv1.1 protein that is truncated in the C-terminal (Kv1.1ΔC79). The patient carrying this mutation suffers from a drug resistant form of EA1 [16].

Truncated Kv1.1 channels have previously been studied in cell culture and *Xenopus *oocytes. When the Kv1.1ΔC79 protein is expressed in cell culture it is trapped in the endoplasmic reticulum (ER) and degraded [17]. However, full length Kv1.1 in cell culture is also retained in the ER [18,19]. Another experimental Kv1.1 protein truncated in the extracellular loop between S1 and S2 is able to assemble with full length Kv1.5 subunits in cell culture and the resulting complexes are trapped in the ER [20]. In *Xenopus *oocytes both the N- and C-terminal truncated Kv1.1 variants have a dominant negative effect on currents when coinjected with full length Kv1.1 and Kv1.2 [21,22]. Knowing if the MCEPH protein is expressed and how it interacts with other Kv1 subunits would provide further understanding of Kv1 trafficking and clues to the downstream effects seen in the *mceph/mceph *mice.

In this paper we show that a truncated Kv1.1 protein, MCEPH is expressed in the brain of the *megencephaly *mouse. The truncated protein is trapped in the ER and does not reach the plasma membrane of neurons. MCEPH has the ability to form multimers with Kv1.1. It has a dominant negative effect on Kv1.2 and Kv1.3 currents in *Xenopus *oocytes, but in the brain it does not appear to retain Kv1.2 in the ER.

## Results

### Antibody characterization

**Figure 1:**
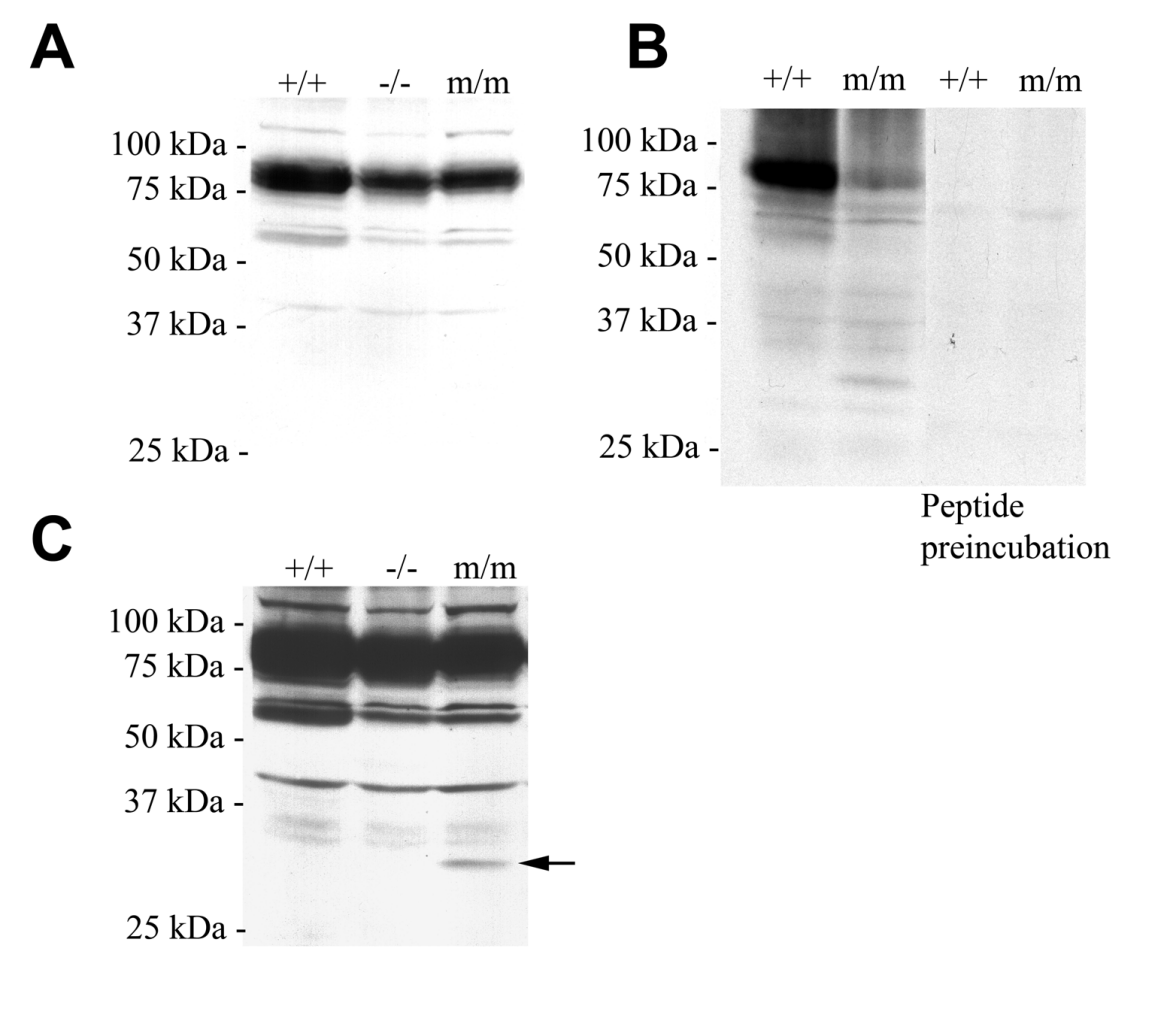
**Immunoblot using the Kv1.1 N-terminal antibody on brain lysate from wild type, Kv1.1-null and *mceph/mceph***. A. Lysate from wild type (+/+), Kv1.1 null (-/-) and *mceph/mceph *(m/m) brains were loaded on SDS-PAGE and immunoblotted with the polyclonal Kv1.1 N-terminal antibody. In wild type lysate a strong band was detected at 86 kDa, corresponding to Kv1.1. The same band was seen in the Kv1.1 null and *mceph/mceph *lysates but at a lower intensity. The 86 kDa bands in Kv1.1 null and *mceph/mceph *lysates are due to antibody cross reactivity since neither Kv1.1 null nor *mceph/mceph *mice have any full-length Kv1.1 protein. B. The polyclonal Kv1.1 N-terminal antibody was preincubated with the peptide used for immunization. Lysate from wild type (+/+) and *mceph/mceph *(m/m) brains were loaded on SDS-PAGE and immunoblotted with the Kv1.1 N-terminal antibody without or after preincubation. The preincubation completely blocked the signal. C. A longer exposure of the immunoblot in panel A. In *mceph/mceph *brain lysate there was a unique band at approximately 30 kDa, which corresponds to the calculated weight of MCEPH (arrow).

A polyclonal antiserum was produced by immunization of rabbits with a synthetic peptide corresponding to amino acids 4 to 27 of Kv1.1. The N-terminal epitope was necessary to be able to detect the truncated MCEPH protein. Western blot on brain lysate from wild type mice using the antiserum showed a strong band at 86 kDa but also several weaker bands. The 86 kDa band had the same size as that detected by the previously characterized monoclonal antibody against a C-terminal epitope of Kv1.1 [23]. Preimmune serum did not recognize the 86 kDa band but showed the same weaker bands as the antiserum. The antiserum was affinity purified against the peptide used for immunization which reduced the number of unspecific bands significantly (Figure 1 panel A). Preincubation of the affinity purified antibody with the peptide used for immunization absorbed the signal (Figure 1 panel B). The purified Kv1.1 N-terminal antibody was used for all further experiments.

In immunblotting of lysate from Kv1.1 null mice an 86 kDa band was present but at a lower intensity than in wild type (Figure 1, panel A). Since the null mouse does not have any Kv1.1 protein this means that the antibody cross reacts with another protein. Kv1 subunits have a high degree of similarity so it is likely that the antibody recognizes another Kv1 subunit. The peptide used for immunization share a stretch of six amino acids with Kv1.2. Both Kv1.1 and Kv1.2 are expressed in the brain as mature glycoproteins that are reported to be 86 and 88 kDa, respectively [23]. The shared amino acids and the size of the band makes Kv1.2 the likely candidate for the cross reactivity.

**Figure 2:**
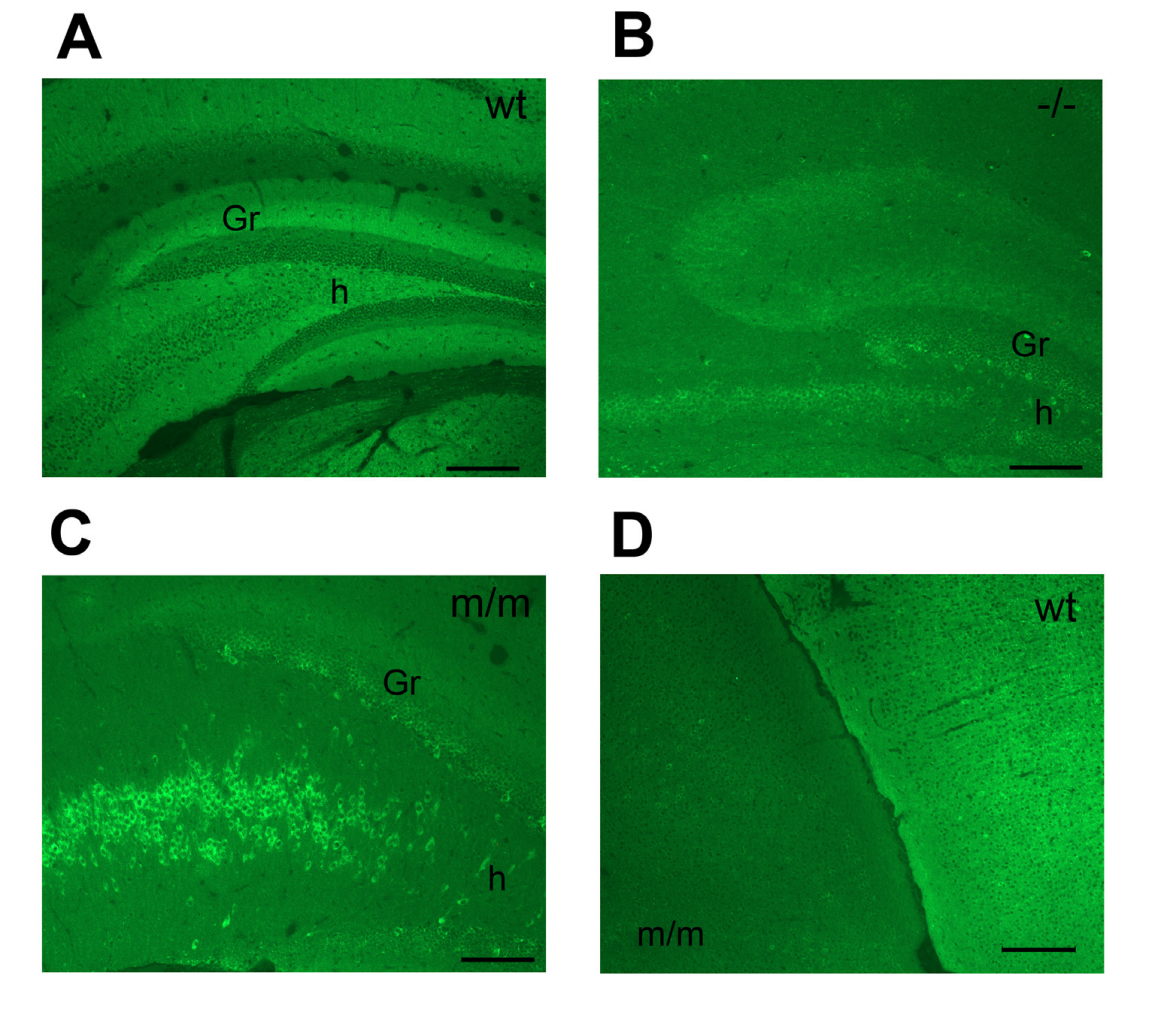
**Kv1.1 immunohistochemistry in wild type, Kv1.1-null and *mceph/mceph *hippocampus**. Immunohistochemistry on formalin fixed brain sections. Exposure parameters were adjusted to obtain a strong and clear signal. Note exposure times given to relate between panels A. Wild type hippocampus showed the same staining pattern as that previously reported for Kv1.1 [2] (exposure 1100 ms) B. Kv1.1 null mouse hippocampus: some cross reactivity of the antibody was seen. (exposure 2700 ms) C. In the *mceph/mceph *hippocampus the immunoreactivity surrounded the nuclei of neurons especially in the dentate gyrus hilus (h) (exposure 2500 ms) D. Parietal neocortex in *mceph/mceph *(left) and wild type (right) brain: the staining of fibers in wild type was absent in *mceph/mceph*. Scale bar 200 μm; h, dentate gyrus hilus; Gr, dentate gyrus granular cell layer; wt, wild type; -/-, Kv1.1-null; m/m, *mceph/mceph*

Acetone post fixed wild type and Kv1.1-null brains were used for immunohistochemistry. In wild type the staining resembled Kv1.1 but the background was high. The pattern in Kv1.1 null brain was very similar to wild type, indicating cross reactivity (data not shown). Immunohistochemistry on formalin fixed wild type brain sections showed the same staining pattern that previously has been reported for Kv1.1 [2]. In the hippocampus the immunoreactivity was mainly localized in the fiber networks in the middle part of the molecular layer, in the hilus and the pyramidal cell layer of CA3 (Figure 2, panel A). When formalin fixed tissue was used the pattern in Kv1.1 null was changed and clearly distinguishable from wild type (Figure 2, panel B).

The conclusion is that the antibody recognizes Kv1.1. It is cross reactive when used for immunoblotting but this is greatly reduced when used for immunohistochemistry on formalin fixed tissue.

### MCEPH expression in the brain

The 11 bp deletion in *Kv1.1 *in *mceph/mceph *mice leads to a premature stop codon. The predicted truncated protein, MCEPH, has a calculated weight of 27 kDa. To determine if MCEPH is expressed in the brain the polyclonal Kv1.1 N-terminal antibody was used for immunoblotting and immunohistochemistry. Immunoblotting showed a band at approximately 30 kDa in brain lysate from *mceph/mceph *mice (Figure 1, panel C). The intensity of the band was much lower than that of the Kv1.1 protein in wild type mice suggesting a low expression of the truncated protein. The *mceph/mceph *mice do not have any full length Kv1.1 but, like the Kv1.1 null mice, they have the 86 kDa cross reactivity-caused band. However, the 30 kDa band was only present in *mceph/mceph *mice. No other band specific for *mceph/mceph *was detected.

Immunohistochemistry was performed on formalin fixed *mceph/mceph *mice brain sections. Interestingly, in *mceph/mceph *there was staining only around the nuclei of cells, and no staining in fibers (Figure 2, panel C and D). There was some background staining resembling that of the Kv1.1 null mouse but the overall pattern was significantly different. The main areas with MCEPH immunoreactivity were the hippocampus and ventral cortex. In the other areas examined there were only a few stained cell bodies. In the hippocampus MCEPH was expressed in neurons in the dentate gyrus, CA1 and in CA3. This pattern is in agreement with the previously reported mRNA expression [2]. Taken together, the immunoblotting and immunohistochemistry results show that MCEPH is expressed in the brain of the *megencephaly *mouse.

### Trafficking of MCEPH

Kv1.1 has a single N-glycosylation site on the extracellular loop between S1 and S2 and is expressed on the plasma membrane as a mature glycoprotein. This glycosylation site is preserved in MCEPH which makes it possible to study the trafficking of MCEPH by analyzing its glycosylation pattern. Glycosylation starts in the ER where a high mannose carbohydrate is transferred to an asparagine residue. This carbohydrate can be cleaved off by the glycosidases EndoH and PNGaseF. Next, the protein is transported to the Golgi where the carbohydrate is modified and becomes resistant to EndoH but not to PNGaseF. This is used as a marker for ER to Golgi transport.

**Figure 3:**
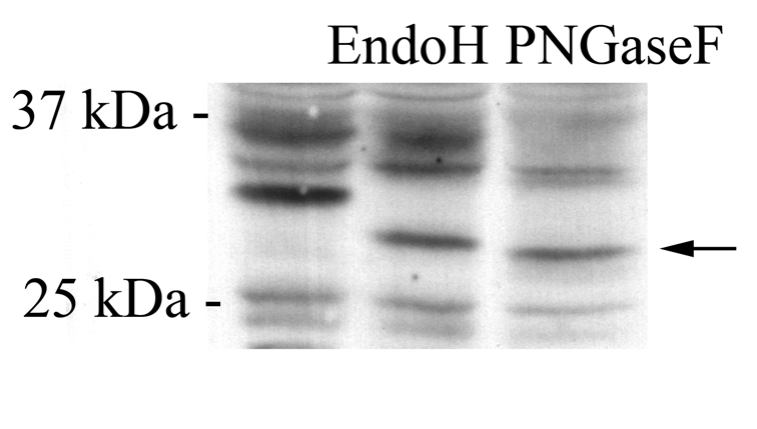
**Trafficking of MCEPH**. Trafficking of MCEPH in the brain was determined by analyzing glycosylation pattern. Both EndoH and PNGaseF reduced the molecular weight with approximately 3 kDa (arrow). This corresponds to core glycosylation. No unglycosylated MCEPH was detected.

To investigate the glycosylation status, brain lysates from wild type and *mceph/mceph *mice were treated with the enzymes EndoH and PNGaseF and analyzed with immunoblotting. For Kv1.1 in wild type mice EndoH did not affect the size of the 86 kDa band but PNGaseF reduced it with approximately 20 kDa. This is in agreement with other studies that have shown that the 86 kDa band is the mature glycosylated form of Kv1.1 expressed in the plasma membrane [18]. Both EndoH and PNGaseF reduced the apparent molecular weight of MCEPH with approximately 3 kDa (Figure 3). This strongly indicates that MCEPH is core glycosylated in the ER but not transported to the Golgi. Neither mature nor unglycosylated MCEPH was detected in the brain.

### Interactions of MCEPH

Kv1 channels are tetramers and the assembly of the Kv1 subunits takes place in the ER [24]. MCEPH contains the N-terminal tetramerization domain and might thus bind other Kv1 subunits.

#### Interactions with Kv1.2 in the brain

**Figure 4:**
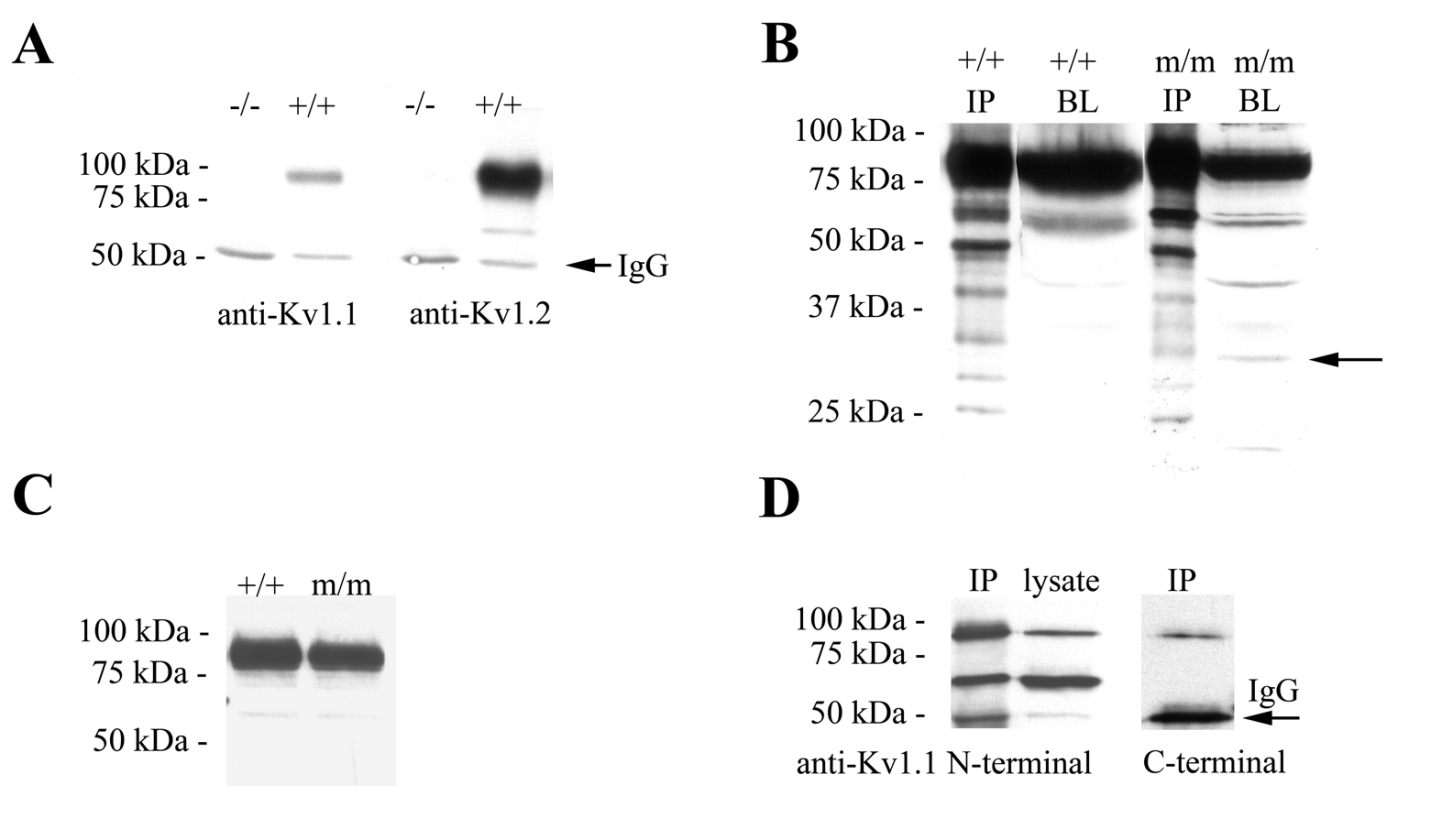
**Analysis of interactions between MCEPH and Kv1 subunits**. A. Immunoprecipitation was performed using a monoclonal Kv1.1 C-terminal antibody on brain lysate from wild type (+/+) and Kv1.1 null (-/-) mice. In wild type brain both Kv1.1 and Kv1.2 are detected. In Kv1.1 null brain neither Kv1.1 nor Kv1.2 was detected. B. Immunoprecipitation was performed with the anti-Kv1.2 monoclonal antibody on brain lysate from wild type (+/+) and *mceph/mceph *(m/m) mice. The immunoprecipitation reaction and corresponding brain lysate was loaded on SDS-PAGE. In brain lysate from *mceph/mceph *(BL) MCEPH was detected using the polyclonal Kv1.1 N-terminal antibody (arrow). However, no MCEPH band was detected in the immunoprecipitate from *mceph/mceph *(IP). C. Hippocampi were dissected and an equal amount of lysate from wild type and *mceph/mceph *was loaded on SDS-PAGE and immunoblotted with anti-Kv1.2. Only a very small fraction of the Kv1.2 was core glycosylated (60 kDa). There appeared to be no increase in the amount of core glycosylated Kv1.2 in *mceph/mceph *hippocampus compared to wild type. D. HEK293 cells were cotransfected with Kv1.1-DsRed and MCEPH-ZsGreen constructs. Both the 85 kDa Kv1.1-DsRed and the 55 kDa MCEPH-ZsGreen fusion proteins were detected with immunoblotting on cell lysate using the polyclonal Kv1.1 N-terminal antibody (lysate). The relative levels of the two fusion proteins in this overepressing cell system cannot be used to quantitate MCEPH expression or stability in brain since regulation and trafficking is known to be different between these two systems. The lysate was immunoprecipitated with the Kv1.1 C-terminal antibody. In the precipitate, both fusion proteins were detected with the Kv1.1 N-terminal antibody (IP). When the Kv1.1 C-terminal antibody was used for immunoblotting only the Kv1.1-DsRed fusion protein was detected.

Kv1.1 and Kv1.2 are often associated in the same channels in the brain [10,25]. We have previously reported a disturbance in the expression of Kv1.2 in the *mceph/mceph *hippocampus [2]. To investigate if this disturbance could be caused by an interaction between MCEPH and Kv1.2, immunoprecipitation followed by immunoblotting was performed on brain lysate from wild type, Kv1.1 null and *mceph/mceph *mice. The monoclonal Kv1.1 and Kv1.2 antibodies each precipitated both Kv1.1 and Kv1.2 in wild type brain, verifying that correct experimental procedures were used. As a control the same antibodies were used for immunoprecipitation of lysate from Kv1.1 null brain. As expected, in the Kv1.1 null lysate no Kv1.1 coprecipitated with Kv1.2. Also, when the Kv1.1 antibody was used for immunoprecipitation neither Kv1.1 nor Kv1.2 was detected (Figure 4 panel A). The Kv1.1-N-terminal antibody precipitated Kv1.1 and Kv1.2 in wild type but also Kv1.2 in Kv1.1 null lysate. This means that it is not specific when used for immunoprecipitation. For *mceph/mceph*, immunoprecipitation was performed with anti-Kv1.2 whereas the Kv1.1-N-terminal antibody was used for immunoblotting. With this approach it was not possible to detect any MCEPH co-precipitating with Kv1.2 (Figure 4 panel B). This suggests that there is no persistent interaction between Kv1.2 and MCEPH in the brain.

On Western blots, Kv1.2 in brain appears as two bands at 60 and 88 kDa representing core and mature glycosylated form, respectively [26]. If MCEPH binds and traps Kv1.2 in the ER, the amount of core glycosylated Kv1.2 would be expected to increase and the mature glycosylated form decrease. Immunoblotting was performed on lysates from both whole brains and isolated hippocampi. The hippocampus was investigated since MCEPH is expressed primarily in this brain region. Most of the Kv1.2 protein in the hippocampus had mature glycosylation and there was only a very small amount of core glycosylated protein. No difference in Kv1.2 core glycosylation was seen between wild type and *mceph/mceph *in neither whole brain nor hippocampus lysate (Figure 4 panel C). When the immunoprecipitation and glycosylation data is combined it appears that MCEPH does not retain Kv1.2 in the ER in brain.

#### Interactions with Kv1.1 in HEK293 cells

To test if MCEPH can form multimers its ability to interact with wild type Kv1.1 was investigated by overexpression in cell culture. This system was chosen to allow higher expression levels and easier detection. For this experiment Kv1.1 and MCEPH were cloned into the vectors pZsGreen and pDsRed2 to construct fluorescent fusion proteins. HEK293A cells were transfected with the MCEPH-ZsGreen and the Kv1.1-DsRed constructs together or separately and analyzed 48 hours after transfection. The green and the red fluorescent signals were colocalized around the cell nucleus (data not shown). No staining was observed on the plasma membrane. Western blot with the polyclonal Kv1.1 N-terminal antibody on lysate from cotransfected HEK293 cells showed bands at 55 and 85 kDa corresponding to the MCEPH and Kv1.1 fusion proteins (Figure 4 panel D). Corresponding bands were found in cells transfected with single constructs which confirmed the identity of the bands. To investigate the interaction between MCEPH and Kv1.1, immunoprecipitation was performed on cell lysate from cotransfected cells using the monoclonal Kv1.1 C-terminal antibody. Immunoblotting with the polyclonal Kv1.1 N-terminal antibody detected the MCEPH-ZsGreen fusion protein as well as the Kv1.1-DsRed fusion protein (Figure 3 panel D). Since the C-terminal Kv1.1 antibody is specific for wild type Kv1.1 the detected MCEPH protein was associated with the Kv1.1 protein.

#### Interactions with Kv1.2 and Kv1.3 in Xenopus oocytes

**Figure 5:**
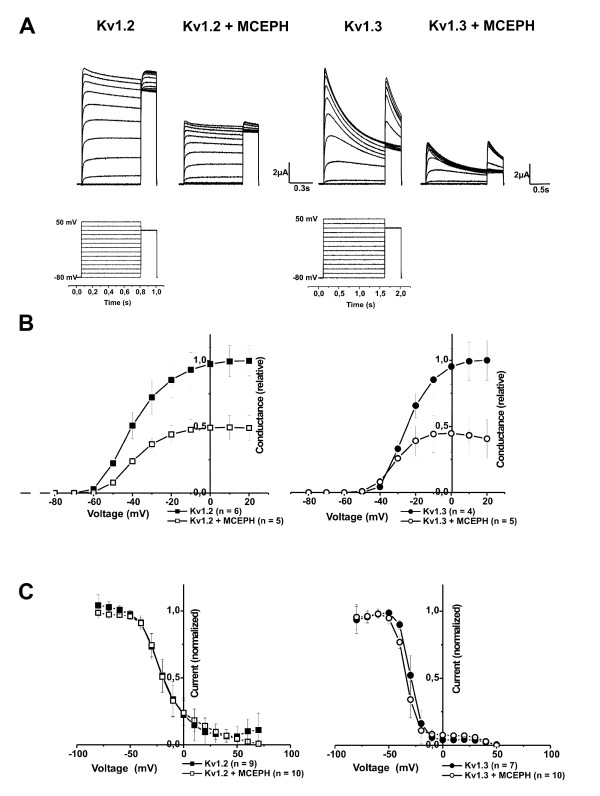
**Electrophysiological recordings demonstrating the interaction between MCEPH and Kv1.2 and Kv1.3 in *Xenopus *oocytes**. A. Currents of Kv1.2 and Kv1.3, when expressed separately and when coexpressed with MCEPH in *Xenopus *oocytes. Pulse steps from -80 to +50 mV (increment between the steps 10 mV), followed by a step to +30 to enable measurements of the inactivation. B. Activation curves (peak conductance versus voltage) for Kv1.2 and Kv1.3, when expressed separately and when coexpressed with MCEPH. Same stimulation protocols as in A. Error bars mark standard error of mean. C. Inactivation curves (normalized peak conductance, associated with the second pulse, versus voltage of preceding step; see panel A) for Kv1.2 and Kv1.3, when expressed separately and when coexpressed with MCEPH. Same stimulation protocols as in A. Error bars mark standard deviation.

To further investigate the ability of MCEPH to interact with Kv1 subunits we chose to analyze the effects of expressing MCEPH in combination with Kv1.2 or Kv1.3 in *Xenopus laevis *oocytes. Figure 5a shows the Kv1.2 and Kv1.3 currents associated with a series of voltage steps, followed by a step to +30 mV. Both channels show delayed rectifier behaviour and lack fast inactivation. Kv1.3, however, shows a marked slow inactivation.

MCEPH was found to reduce the expression of both Kv1.2 and Kv1.3 in the plasma membrane. Figure 5b shows the mean results of injecting *mceph *mRNA together with *Kv1.2 *or *Kv1.3 *mRNA (in equal amounts) given as the effect on peak conductance versus voltage curves (i.e activation curves). As seen, the curves for Kv1.2 and Kv1.3 are down-scaled by about 50 and 60%, respectively, without marked changes in slope or midpoint value. The MCEPH shift of the Kv1.3 activation curve was slightly bigger than that of Kv1.2 (-6 mV vs. +2 mV; Table 1). The activation time course of the currents associated with voltage steps was negligibly affected by MCEPH, reflected in the unaffected time to peak current at +50 mV for both Kv1.2 and Kv1.3 (Table 1). Similar effects were observed for the MCEPH effect on the slow inactivation kinetics. Mean inactivation versus voltage curves, measured from the peak current associated with the second pulse (Figure 5a) are shown in Figure 5c. The MCEPH shift of the Kv1.3 inactivation curve was slightly bigger than that of Kv1.2 (-5 mV vs. -1 mV; Table 1).

In summary, the measurements show that MCEPH has a dominant negative effect on both Kv1.2 and Kv1.3 channel expression in *Xenopus *oocytes. Furthermore, the small effects on activation and inactivation kinetics suggest that MCEPH does not form functional channel complexes with Kv1.2 or Kv1.3 subunits in the plasma membrane. Heteromeric complexes involving MCEPH subunits are expected to drastically affect channel kinetics.

## Discussion

In this study we show that *mceph/mceph *mice express a truncated Kv1.1, MCEPH. The MCEPH protein is expressed in neurons throughout the brain but is trapped in the ER. MCEPH is able to bind full length Kv1.1 in cell culture and has a dominant negative effect when coexpressed with Kv1.2 and Kv1.3 in *Xenopus *oocytes. In contrast, in the brain MCEPH does not appear to retain Kv1.2 in the ER.

To be able to detect the truncated MCEPH protein an antibody against the N-terminal part of Kv1.1 was generated. Immunoblotting on brain lysate from *mceph/mceph *mice showed a band that has the same size as the predicted MCEPH protein, approximately 30 kDa. This band is present in all *mceph/mceph *brains studied but not in any wild type or Kv1.1 null mice. Based on this we conclude that MCEPH is expressed in the brain of the *megencephaly *mouse.

Kv1.1 in the brain is a mature glycoprotein that is mostly localized to axons and nerve terminals [6]. Truncated Kv1.1 variants have previously been shown to be retained in the ER in cell culture [17,20]. The draw back of these studies is that full length Kv1.1 homomers in cell culture is also largely retained in the ER since they are undetectable at the cell surface by immunohistochemical methods [19] but detectable at low levels with electrophysiological methods [27]. Lack of surface expression was seen by us both in HEK293 cells and in the neuron-like cell line PC12 (data not shown). The reason is a strong ER retention motif in the pore region [28]. The *mceph/mceph *mouse provides an opportunity to study the trafficking of a truncated Kv1.1 in neurons. Both EndoH and PNGaseF treatment reduced the molecular weight of MCEPH by approximately 3 kDa, suggesting that the protein is core glycosylated [18]. We detected neither maturely glycosylated nor unglycosylated MCEPH. The glycosylation data suggest that MCEPH is trapped in the ER and not further transported. This is supported by immunohistochemistry experiments were MCEPH immunoreactivity surrounds the nuclei in an ER-like pattern. There is no immunoreactivity in fibers consistent with the lack of mature glycosylated MCEPH. The low amount of MCEPH protein detected with immunoblotting compared to the high levels of mRNA suggests that the MCEPH protein is rapidly degraded. This is most likely due to ER-associated degradation (ERAD), a quality control mechanism in the ER that recognizes misfolded proteins [29]. These proteins are retro-translocated to the cytosol and degraded by proteasomes.

We have previously seen disturbances of staining pattern of Kv1.2 and Kv1.3 in the *mceph/mceph *hippocampus, which was not due to transcriptional regulation [2]. Previous studies of other truncated K1.1 variants have shown that they have a dominant negative effect in oocytes and form oligomers with full length Kv1 subunits in cell culture. We hypothesized that MCEPH interacts with Kv1 subunits and causes the expression disturbance possibly by retaining these subunits in the ER. Kv1.1 often forms heterotetramers with Kv1.2 subunits in the brain [10,25]. Nevertheless, we found no evidence for a persistent interaction between MCEPH and Kv1.2 in the brain, suggesting that there is no retention of Kv1.2 in the ER. However, we found MCEPH to be able to oligomerize when expressed in HEK293 cells. Moreover, electrophysiological measurements on *Xenopus *oocytes showed that MCEPH reduces both Kv1.2 and Kv1.3 currents, suggesting interaction between MCEPH and Kv1 subunits in this cell type. This indicates that the lack of ER retention seen for Kv1.2 in the brain is not caused by an inability of MCEPH to form multimers

It can be assumed that lack of Kv1.1 could lead to defect trafficking of Kv1 channels. This is not the case since the Kv1.1 null mouse has normal expression of Kv1.2 in the hippocampus (data not shown) and also the cerebellum and sciatic nerve [12,30]. One possibility is that the Kv1.2 and Kv1.3 expression disturbances in *mceph/mceph *hippocampus are related to the growth. The *mceph/mceph *hippocampus is significantly enlarged and has an increased amount of neurons (unpublished data). This could lead to disturbed organization of the dendritic trees and give rise to the abnormal expression patterns. Also, since MCEPH contains the N-terminal T1 domain, which has been shown to mediate beta subunit association [31,32], it is possible that MCEPH sequesters these auxiliary subunits within the ER and prevents them from forming complexes with Kv1.2 and Kv1.3 in *mceph/mceph *mice. Beta subunit association has been shown to promote Kv1 trafficking to the cell surface as well as to cause axonal targeting of Kv1.2 [33].

The *megencephaly *mouse is not a model for any known human disease. Still, it provides a unique tool to study Kv1 channels in the brain. The EA1 mutation R417Stop gives rise to a Kv1.1 protein that lacks the final 79 amino acids in the C-terminal domain (Kv1.1ΔC79). The Kv1.1ΔC79 protein and MCEPH have some similarities but there are major differences. Both truncated proteins have a dominant negative effect in oocytes and can assemble with full length subunits in cell culture. For the Kv1.1ΔC79, change in current kinetics when coinjected with Kv1.1 in *Xenopus *oocytes indicates that some channels containing truncated subunits can reach the plasma membrane [16]. This is not seen for MCEPH which is most likely due to the difference in length. Kv1.1ΔC79 might evade the rapid degradation by cellular control mechanisms since most of the protein remains. The truncated protein can then cause more damage by retaining subunits or by forming defect channels. This is supported by the inheritance patterns. The R417Stop mutation is dominant which suggests a new function for the truncated protein, while *mceph *is recessive, which indicates a loss of function.

The mechanism behind the brain growth in *mceph/mceph *is still unknown. We now know that MCEPH is expressed in the brain, but we do not know if it is MCEPH or lack of functional Kv1.1 that is responsible for the overgrowth. The Kv1.1 null mouse has not been reported to have an enlarged brain which suggests that MCEPH has an effect. However, the low levels of protein and recessive inheritance indicates that the *mceph *mutation causes a loss of function of Kv1.1. In line with both scenarios, it was previously shown that potassium channel dysfunction appears to reduce apoptosis [11], which might contribute to the overgrowth of the *mceph/mceph *brain.

## Conclusion

For the first time we report the expression of a truncated Kv1.1 protein in brain. This protein is trapped within ER and has the ability to interact with Kv1 subunits. The *megencephaly *mice, where the protein is expressed, constitute a tool to study Kv1.1 trafficking relevant for understanding epilepsy, ataxia and pathologic brain overgrowth.

## Methods

### Antibodies

#### Primary antibodies

A rabbit polyclonal antibody against the N-terminal of Kv1.1 was generated using a synthetic peptide (CMSGENADEASTAPGHPQDGSYPRQ) corresponding to amino acids 4–27. Kv1 subunits have a high degree of homology. The peptide for immunization was selected to have as low similarity as possible to other Kv1 subunits. The peptide was conjugated to KLH and injected into rabbits for production of antiserum. The antiserum was affinity purified against the peptide using standard procedures. The affinity purified antibody was used at a 1:500 dilution for immunoblotting and at 1:400 for immunohistochemistry. Monoclonal anti-Kv1.1 and anti-Kv1.2 (Upstate Biotechnologies Inc, Waltham, MA, USA) were used for immunoblotting at dilutions of 1:200 and 1:1000, respectively.

#### Secondary antibodies

For immunoblotting, horseradish peroxidase coupled swine anti-rabbit (DAKO, Glostrup, Denmark 1:2000) and goat anti-mouse (Upstate Biotechnologies Inc 1:5000) were used. For immunohistochemistry a FITC labeled goat anti-rabbit antibody (Sigma-Aldrich, St. Louis, MO, USA 1:300) was used.

### Experimental animals and preparation of tissues

All studies were performed in accordance with guidelines from the Swedish National Board for Laboratory Animals. The BALB/cByJ-*mceph/mceph*, BALB/cByJ-+/+ (wild type) and C3HeB/FeJ-Kcna1^tm1Tem ^(Kv1.1 null) [12] mouse strains were obtained from The Jackson Laboratory (Bar Harbor, ME, USA). Animals were euthanized by CO_2_, and the brains were removed and immersed in ice-cold phosphate-buffered saline (PBS) and immediately thereafter frozen on dry ice. Serial 14 μm sections were cut in a cryostat (Jung CM 3000, Leica Instruments GmbH, Nussloch, Germay) at -15° and thaw-mounted on microscope glass slides (Probe On, Fischer Scientific, Pittsburgh, PA, USA).

For immunohistochemistry, mice were anesthetized with isoflurane and perfused via the ascending aorta with Ca^2+^-free Tyrode's solution followed by a fixative containing 4.0% paraformaldehyde and 0.4% picric acid in 0.16 M phosphate buffer. Brains were dissected out, immersed in ice-cold fixative for 90 min and then rinsed in 0.1 M phosphate buffer (pH 7.4) containing 10% sucrose, 0.01% sodium azide and 0.02% bacitracin (Sigma-Aldrich). Serial 14 μm sections were cut in a cryostat and mounted on gelatin/chrome-alum coated slides.

For immunoprecipitation and immunoblotting, brains were homogenized in RIPA buffer (150 mM NaCl, 50 mM Tris pH 8,0, 1% NP-40, 0,1% SDS, 0,5% sodium deoxycholate) with a protease inhibitor cocktail (Sigma-Aldrich) and centrifuged at 1000 × g to pellet debris.

### Plasmids

The complete Kv1.1 mRNA sequence [NCBI: NM_010595] was PCR amplified from genomic DNA from *mceph/mceph *and *+/+ *mice using High Fidelity Taq polymerase (Roche Diagnostics, Basel, Switzerland). The PCR products were TA-cloned into pCR2.1 (Invitrogen, Carlsbad, CA, USA). The coding sequence was then subcloned into the oocyte expression vector pGEM-HE (Protinac GmbH, Hamburg, Germany) using the HindIII sites at position 823 and 2712. Correct orientation of the insert was confirmed by restriction analysis and sequencing. Rat cDNA for Kv1.2 and Kv1.3 in the pGEM-HE vector was obtained from Protinac.

For expression in mammalian cells the coding sequence of Kv1.1 was PCR amplified from genomic DNA from *mceph/mceph *and *+/+ *mice using two different reverse primers that removed the stop codons. The PCR products were cloned into the vectors pDsRed2-N1 and pZsGreen-N1 (BD Biosciences Clontech Palo Alto, CA, USA) to construct fluorescent fusion proteins.

### Cell culture and transfection

Human embryonal kidney cells HEK293A were cultivated in Dulbecco's modified Eagle's media (DMEM) supplemented with L-glutamine, 10% fetal bovine serum (Gibco, Rockville, MD, USA) and 100 μg/ml penicillin/streptomycin (Gibco) in a 37°C incubator containing 5% CO_2 _humidified air. For transfection, HEK293 cells were plated on 100 mm dishes. At 80% confluency the cells were transfected using Lipofectamine and Plus reagent (Invitrogen) according to the manufacturers protocol and analyzed 48 hours after transfection. The HEK293A cells were lysed in RIPA buffer with a protease inhibitor cocktail (Sigma-Aldrich). The lysate was spun 1000 × g to pellet debris and used for immunoprecipitation and immunoblotting.

### Immunoblotting, immunoprecipitation and glycosylation analysis

For immunoblotting, brain or cell lysate was diluted with 2× Laemmli loading buffer with β-mercaptoethanol and loaded on 10% SDS-PAGE with 4% stacking gel. Proteins were transferred to nitrocellulose membranes (Hybond-ECL, Amersham Biosciences, Little Chalfon, UK). The membranes were blocked in 5% milk and incubated with the primary antibody. This was followed by incubation with a horseradish peroxidase coupled secondary antibody. ECL Western blotting reagent (Amersham Biosciences) was used for detection according to the manufacturer's protocol.

For immunoprecipitation, brain lysate was incubated with 4 μg of the indicated antibody for 2 hours on ice. Protein-A coupled sepharose beads (Amersham Biosciences) were added and the mixture was incubated for 1 hour. The beads were spun down and the supernatant was removed. The beads were then washed three times with RIPA buffer and once with 50 mM Tris pH 8.0. They were then resuspended in Laemmli loading buffer and boiled for 5 min. The supernatant was collected and analyzed by immunoblotting.

For glycosylation analysis, brain tissue was homogenized in a lysis buffer containing 50 mM Tris pH 8.0, 150 mM NaCl, 1 mM EDTA, 1% Triton X and protease inhibitors. The homogenate was spun at 1000 × g to pellet debris. The lysate was denatured with 0.1% SDS and 1% β-mercaptoethanol at 100° for 5 min. Samples were incubated with EndoH or PNGaseF according to the manufacturers protocol (New England Biolabs, Beverly, MA, USA) at 37° for 2 hours and analyzed with immunoblotting.

### Immunohistochemistry

Brain sections were thawed quickly. Fresh frozen sections were fixed in ice cold acetone. All slides were blocked in PBS with 5% goat serum and 0.23% Triton-X. They were then incubated with the primary antibody diluted in blocking solution followed by incubation with a FITC labeled secondary antibody. The slides were mounted with Vectashield antifade mounting media (Vector laboratories, Burlingame, CA, USA) and examined under an Axioskop 2 microscope (Zeiss, Oberkochen, Germany).

### Electrophysiology

The electrophysiological experiments comprised voltage clamp measurements on oocytes from *Xenopus laevis*, injected with *mceph*, *Kcna2 *and *Kcna3 *mRNA. Plasmids with the studied genes were linearized with restriction enzymes *HindIII *(*Kv1.2 *and *Kv1.3*) and *Nhe1 *(*mceph*). The linearized DNA was purified and transcribed with the Message Machine Kit (Ambion, Austin, TX, USA). The mRNA product was purified and dissolved in 10 μl ddH_2_O and stored at -70°C until injection in oocytes. The mRNA injected in oocytes was diluted to one tenth of the original concentration (2.4 mg/ml and 0.8 mg/ml, respectively). The oocytes (in stage V and VI) were surgically removed from the frog under anaesthesia, and treated with Liberase (0.25 mg/ml) for three hours. After being carefully rinsed the oocytes were incubated overnight in modified Barth's solution (88 mM NaCl, 1 mM KCl, 2.4 mM NaHCO_3_, 0.015 mM HEPES, 0.33 mM Ca(NO_3_)_2_, 0.41 mM CaCl_2_, 0.82 mM MgSO_4_, pH adjusted to 7.5) with added 10 μg/ml pyruvate and 10 μg/ml penicillin-streptomycin at 10°C. They were injected with cRNA (50 nl/cell) using a Nanoject injector; Drummond Scientific, Broomall, PA) and incubated at room temperature (20–21°C) 16–24 hours before starting the electrophysiology experiments (day 3 to 4 after injection).

The electrophysiological experiments were performed with a two-electrode voltage-clamp (CA-1 amplifier, Dagan, Minneapolis, MN). Microelectrodes were made from borosilicate glass capillaries with a mechanical puller and filled with 3 M KCl solution, resulting in a resistance between 0.5 to 1.0 MΩ. The extracellular solution was composed of 88 mM NaCl, 1 mM KCl, 0.8 mM MgCl_2_, 0.4 mM CaCl_2_, 15 mM HEPES with pH is adjusted to 7.4. All measurements were carried out at room temperature (20–22°C). The holding potential was set to -80 mV and the interval between the test steps was 2 s (Kv1.2) and 30 s (Kv1.3). The recorded current was filtered by a low-pass Bessel filter (5 kHz) and sampled with intervals of 2 ms (Kv1.2) and 4 ms (Kv1.3). The software used for data collection and -analysis were pClamp5 and Clampfit 8.2 (Axon Instruments Inc., Union City, CA) and Origin 6.0 (Microcal Software Inc., Northampton, MA). To avoid different channel density levels due to trafficking during the electrophysiological experiments we restricted the measurements to a four-hour period.

The conductance (G) was calculated from the current (I_K_) and the associated voltage step (V) by

G = I_K_/(V + 80 mV).     (1)

The conductance normalized to the maximal conductance under control conditions (G_max CTRL_) were fitted to the Boltzmann equation

G/G_max CTRL _= 1/[1 + exp((V - V_1/2_)/s)],     (2)

where V_1/2 _is the potential at half-maximal conductance, i.e. midpoint potential, and s is the slope. Significance of differences was tested using the *t*-test.

## Authors' contributions

SP, ASP, MS and CL initiated the study. ASP planned and performed the cloning, cell culture work, immunohistochemistry, immunoprecipitation, western blot and drafted the manuscript. MA performed and evaluated the immunohistochemistry. SP assisted in the cell culture work. GK performed most of the oocyte work, comprising expression and electrophysiology and drafted the electrophysiological section of the manuscript. KS, JN and PÅ participated in the design, performance and analysis of the oocyte experiments, and developed the oocyte section of the manuscript. CL designed and coordinated the study.

All authors edited the draft and approved the final version of the manuscript.

## Figures and Tables

**Table 1:** Effects of MCEPH on Kv1.2 and Kv1.3 kinetics in *Xenopus *oocytes. Mean midpoints of activation and inactivation curves (V_1/2_) and mean half time values (t_1/2_) for Kv1.2 and Kv1.3 (±SD), when expressed separately and when coexpressed with MCEPH. n is the number of oocytes. The activation and inactivation curves were constructed from measurements based on the stimulation protocols in Fig. 5. Half time value (t_1/2_) was measured as time to half peak current at a 50 mV pulse step (±SD).

	Steady-state activation		Steady-state inactivation	n
		
	V_1/2 _(mV)	t_1/2 _(ms)	V_1/2 _(mV)	
Kv1.2	-24 ± 4	3 ± 1	-17 ± 7	9
Kv1.2 + MCEPH	-22 ± 3	3 ± 1	-18 ± 6	10
Kv1.3	-25 ± 4	6 ± 3	-29 ± 3	7
Kv1.3 + MCEPH	-31 ± 4	6 ± 3	-34 ± 4	10
